# College Faculties

**Published:** 1886-07

**Authors:** 


### College Faculties.

From the announcements of the two medical colleges just received, we learn of several important changes in the medical faculty of Niagara University. On account of ill-health, Prof. W. S. Tremaine has resigned his chair as Professor of the Principles and Practices of Surgery, etc. Prof. Tremaine has been appointed Prof. Emeritus and Consulting Physician to the Sisters' Hospital. His services, both in the college and in the re-organization of the Hospital, have been invaluable. He was greatly beloved by the students,and it was with great regret that his resignation was accepted. To succeed such a man,and to come up to the standard set by him, will be a difficult task ; but, in the selection which has been made, the faculty firmly believe they have found a man who will fill the position. The trustees have elected Dr. W. A. Wheeler, U. S. Marine Hospital Surgeon at this port, to fill the vacancy. The doctor has resided here about a year, and is favorably known to the profession of Buffalo. It is a generally accepted fact that seek where they might, the college could not have made a better selection. The instruction of students is no new thing to Dr. Wheeler, for he has occupied the chair of anatomy in another school in this country. He is a graduate of Harvard College, received his medical education at the Medical College of Maine, and the College of Physicians and Surgeons in New York. He received the degree of doctor of medicine from both these institutions. After some years of practice, he entered the U. S. Service five years since. He has been located at Chicago, Charlestown and other important stations. Both in the service and the profession where he has been stationed, he has taken a high rank. When the position became vacant, attention was naturally turned to him, and his services secured. He is a surgeon of skill and ability. There is no doubt but that the Doctor will add honor to the faculty with which he is now associated, and ably fill the chair to which he has been elected.

The Chair of Anatomy having been resigned by Dr. Heath, the trustees have appointed Dr. C. M. Daniels to fill the vacancy. This is a most excellent selection, and all agree that anatomy will be well and faithfully taught in the school.

Dr. F. S. Crego, for several years lecturer in this school, has been appointed Professor of Nervous and Mental Diseases. Dr. W. H. Pitt has been appointed Professor of General Chemistry. Both of these are well-earned promotions.

Thomas W. Lothrop, a graduate of Tufft's College, Massachusetts, a chemist of recognized ability, has been appointed Demonstrator of Chemistry. Mr. Lothrop held this position for a time last year, and proved himself an able teacher.

The faculty of the Buffalo College remains the same, with the exception of the changes noted in a previous number.

